# Effect of the *res2* transcription factor gene deletion on protein secretion and stress response in the hyperproducer strain *Trichoderma reesei* Rut-C30

**DOI:** 10.1186/s12866-023-03125-z

**Published:** 2023-11-30

**Authors:** Jawad Alharake, Frédérique Bidard, Thiziri Aouam, Catherine Sénamaud-Beaufort, Antoine Margeot, Senta Heiss-Blanquet

**Affiliations:** 1 IFP Energies Nouvelles, 1 et 4, avenue de Bois-Préau, Rueil-Malmaison Cedex, 92852 France; 2 Département de biologie, GenomiqueENS, Institut de Biologie de l’ENS (IBENS), CNRS, INSERM, Université PSL, École normale supérieure, Paris, 75005 France

**Keywords:** Fungi, *Trichoderma reesei*, Cellulases, Secretion, Secretion stress, UPR

## Abstract

**Background:**

The fungus *Trichoderma reesei* is one of the most used industrial cellulase producers due to its high capacity of protein secretion. Strains of *T. reesei* with enhanced protein secretion capacity, such as Rut-C30, have been obtained after several rounds of random mutagenesis. The strain was shown to possess an expanded endoplasmic reticulum, but the genetic factors responsible for this phenotype remain still unidentified. Recently, three new transcription factors were described in *Neurospora crassa* which were demonstrated to be involved in protein secretion. One of them, RES2, was involved in upregulation of secretion-related genes. The aim of our present study was therefore to analyze the role of RES2, on protein secretion in the *T. reesei* Rut-C30 strain.

**Result:**

Deletion of the *res2* gene in Rut-C30 resulted in slightly slower growth on all substrates tested, and lower germination rate as well as lower protein secretion compared to the parental strain Rut-C30. Transcriptomic analysis of the Rut-C30 and the Δ*res2* mutant strain in secretion stress conditions showed remarkably few differences : 971 genes were differentially expressed (DE) in both strains while 192 genes out of 1163 (~ 16.5%) were DE in Rut-C30 only and 693 out of 1664 genes (~ 41.6%) displayed differential expression solely in Δ*res2*. Notably, induction of protein secretion by cultivating on lactose and addition of secretion stress inducer DTT induced many genes of the secretion pathway similarly in both strains. Among the differentially expressed genes, those coding for amino acid biosynthesis genes, transporters and genes involved in lipid metabolism were found to be enriched specifically in the Δ*res2* strain upon exposure to lactose or DTT. Besides, redox homeostasis and DNA repair genes were specifically upregulated in the Δ*res2* strain, indicating an altered stress response.

**Conclusion:**

These results indicate that in the *T. reesei* Rut-C30 strain, RES2 does not act as a master regulator of the secretion pathway, but it contributes to a higher protein secretion by adjusting the expression of genes involved in different steps of protein synthesis and the secretion pathway.

**Supplementary Information:**

The online version contains supplementary material available at 10.1186/s12866-023-03125-z.

## Background

 The filamentous fungus *Trichoderma reesei* is a well-known enzyme producer, and hypersecreting strains are industrially used to produce cellulases. One of the best studied hyperproducing strains is Rut-C30 that has been derived from the wild type (WT) strain QM6a by several rounds of mutagenesis. Sequencing of both strains has revealed a total of 269 single nucleotide polymorphisms (SNPs), three chromosomal translocations, one inversion, eight small insertions or deletions (indels), and five large deletions in Rut-C30 [1–3]. Moreover, *cre1* is truncated at its 3’ end with a loss of 2478 base pairs resulting in a low basal cellulase expression in the presence of D-glucose [4]. Rut-C30 is also unable to efficiently grow and produce enzymes on α-linked glucans [5].

In addition to having efficient systems for transporting carbon sources and highly inducible cellulase genes, mutant *T. reesei* strains, such as Rut-C30 also possess a well-developed secretion system. Rut-C30 was found to secrete 2.7 times more proteins than QM6a in 6% (w/v) roll-milled cotton [6]. Using transmission electron microscopy (TEM), several studies investigated the ultrastructure of *T. reesei* [7–9]. They found a link between the high protein secretion capacity of Rut-C30 and the changes in its ultrastructural characteristics in relation to the wild type QM6a. On a microcrystalline cellulose culture, an approximately seven-fold higher ER content in Rut-C30 than in QM6a was observed during the secretory phase, i.e. during the third and sixth day of culturing. Unlike QM6a that had short and vesicular or narrow intracisternal ER profile, Rut-C30 was distinguished by its long and stacked ER whose cisternal space was wide and filled with amorphous material [7]. Similarly, a culture of QM6a and Rut-C30 on microcrystalline cellulose resulted in an about three-fold transient increase in the ER surface area in Rut-C30 after 96 h of culture in comparison to QM6a [8]. In addition, [9] demonstrated that distinct ultrastructural features of Rut-C30 in the early secretory pathway (24 h of cellulase induction) are inherent to the strain rather than a response to increased protein secretion. Despite the sequencing of Rut-C30 and QM6a, there is still no straightforward explanation for these ultrastructural characteristics of Rut-C30.

The secretion system has been well studied in other organisms such as *S. cerevisiae*, *Aspergillus* species and higher eukaryotes [10]. In fungi, most secreted proteins follow the ER-Golgi secretion pathway. After polypeptides are synthesized in the ribosomes, they are processed in the endoplasmic reticulum (ER) and then in the Golgi-apparatus before being secreted into the extracellular medium via secretory vesicles [10, 11]. Certain conditions, however, such as high load of protein production, inhibit the proper folding of proteins resulting in secretion stress. In response to secretion stress, several mechanisms are activated, mainly the unfolded protein response (UPR), UPR-linked ER-associated degradation (ERAD) pathway, and repression under secretion stress (RESS) response [12]. UPR is a highly conserved intracellular signaling pathway between the ER and the nucleus, and it involves two main regulators: ER-transmembrane protein IRE1 and a bZIP transcription factor HAC1. Upon secretion stress, HAC1 is activated, leading to its migration to the nucleus and binding to the unfolded protein response elements (UPREs) found in the promoters of certain genes, such as the ER-chaperon *bip1* and the foldase *pdi1*, which reinforce ER folding capacity [13]. Interestingly, various mechanisms exist for the regulation of UPR in fungi. Some of the mechanisms are HAC1-independent, as found in *A. niger*, [14] while others are IRE1-independent, as it is the case in yeast [15, 16] where TFs different than HAC1, such as Gcn4p, might induce the expression of UPR target genes [17]. In *T. reesei*, the activation of UPR in an IRE1/HAC1-dependent manner has been evidenced upon treatment with DTT and in heterologous protein producing strains 13]. DTT induces UPR by preventing the formation of disulfide bonds which is required for the proper folding of proteins and their transport from the ER [12]. UPR was also triggered during cellulase secretion in lactose containing media [18]. In cDNA subtraction libraries, more than 400 genes including previously known UPR-related genes were found to be induced under secretion stress [13]. But it is presently not known if also other regulators are involved in the control of this secretion stress response, and if these could be responsible for the hyperproducing phenotype of Rut-C30.

In *N. crassa*, three TFs (RES-1, RES2, and RRG-2) have been identified to play a role in cellulase and hemicellulase secretion. Unlike Δ*res-1*, Δ*res2* and Δ*rrg-2* strains showed nearly 30% decrease of cellulase secretion in comparison to the WT [19]. Three- to fourfold reduction in expression of genes coding for hemicellulases was observed in addition to 1.6–1.8-fold decrease in *cbh-1* and *cbh-2* genes. Some of the differentially expressed genes in the Δ*res2* strain relative to the WT have roles in cellular export and secretion in addition to cellular import (endocytosis) which contribute to hyphal tip growth and tip secretion. It was thus suggested that RES2 regulates secretory pathways in *N. crassa* [19].

To elucidate the role of the RES2 homolog in *T. reesei* in secretion and the secretion stress response, the corresponding gene was deleted in the hyperproducing strain Rut-C30. The effect on growth and protein secretion was assessed and a transcriptomic analysis was conducted under two secretion stress conditions: cellulase induction by lactose and addition of dithiothreitol (DTT). Results show that RES2 does not have the same importance for protein secretion in *T. reesei* Rut-C30 as in *N. crassa* but exerts a more subtle regulatory function on protein synthesis, the secretion pathway and stress response.

## Results

### The *T. reesei* Δ *res2* mutant shows growth and germination defects

The *res2* ortholog in *T. reesei* was identified with fungiDB. Pair-wise sequence alignment with the *N. crassa* RES2 showed a 60% sequence identity on the amino acid level between both sequences. To study the role of the *res2* orthologue of *T. reesei*, the gene was deleted using CRISPR-Cas9. After genetic validation by PCR and sequencing, confirming the correct insertion of the deletion cassette, three of the validated transformants were randomly kept for further analysis. The absence of off-target integrations was also confirmed in these mutants by qPCR.

**Fig. 1:**
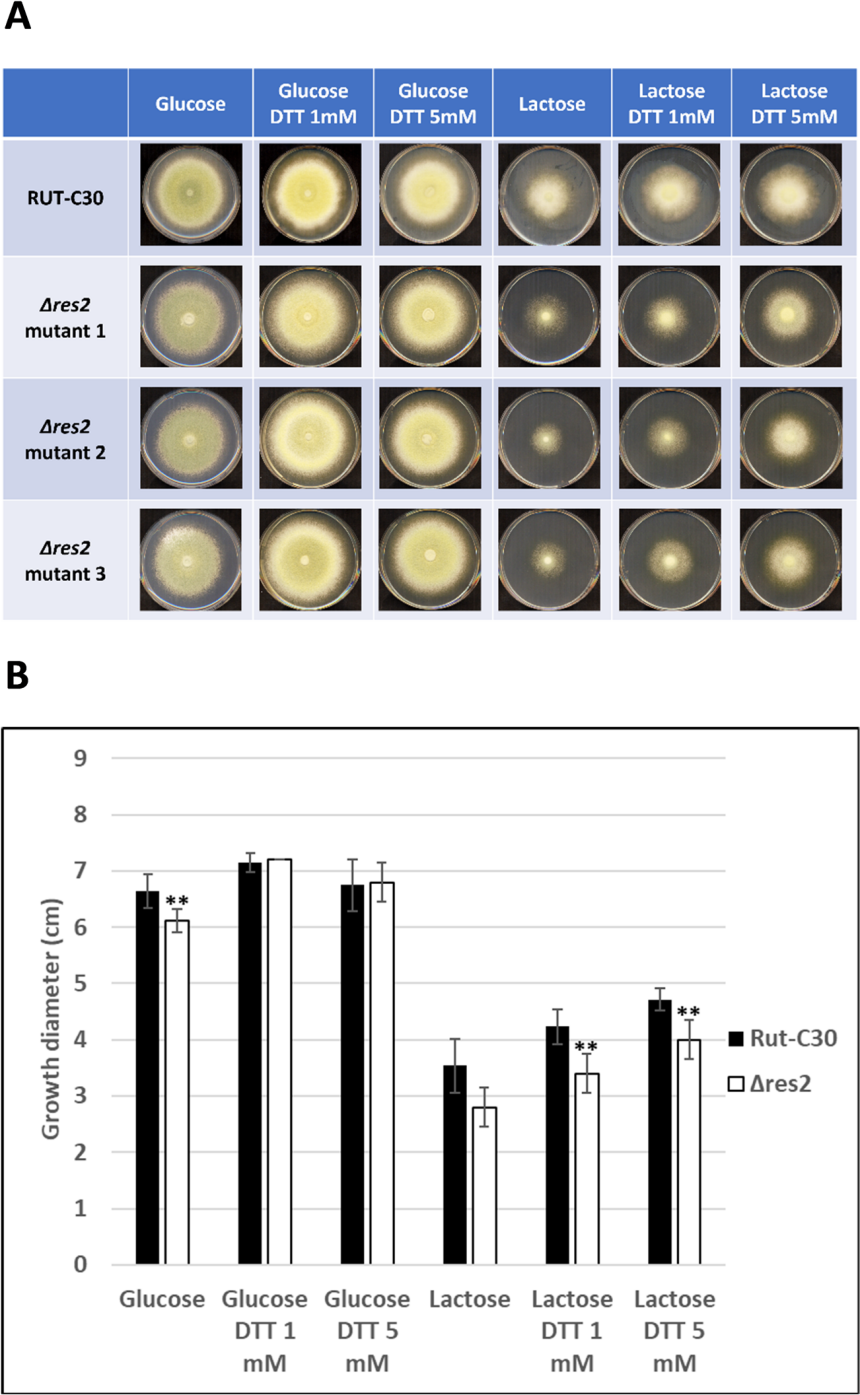
Growth of three ∆res2 transformants and Rut-C30 on different substrates.** A **Rut-C30 and Δres2 transformants were grown on minimal medium supplemented with 2% non-inducing substrate (glucose) or 2% inducing substrate (lactose), with or without addition of DTT. Photos and measurements were done on the fifth day after inoculation. For each strain, three biological replicates were assayed, but only one replicate is shown representatively. **B** Size of colonies of the ∆res2 mutant compared to its parental strain Rut-C30. The error bars indicate the standard deviation of three biological replicates. Significance of the difference between the Rut-C30 and the mutant strains was calculated with the two-tailed t-test with independent variables (**P < 0.01)

Although deletants showed no phenotype on transformation plates, we first wanted to investigate if the *res2* deletion impacts basic functions such as growth and germination. To address this question, Rut-C30 and the three deletion transformants were plated on solid minimal medium containing glucose or a cellulase secretion-inducing substrate such as lactose in the presence or absence of 1 or 5 mM DTT. Figure 1A shows that growth of both Rut-C30 and the mutant was faster on glucose and slower on lactose. However, growth of the *∆res2* mutant was clearly reduced compared to that in the Rut-C30 parental strain on both substrates, but especially on lactose, Fig. 1A. It is interesting to note that the mutant tended to grow better in the presence of glucose + DTT, reducing the difference with Rut-C30 (Fig. 1B). One hypothesis to explain this observation is that the reducing activity of DTT enhances the activity of some enzymes, as it is the case for cellobiase [20], which leads to increased availability of nutrients and increased fungal growth.

**Fig. 2:**
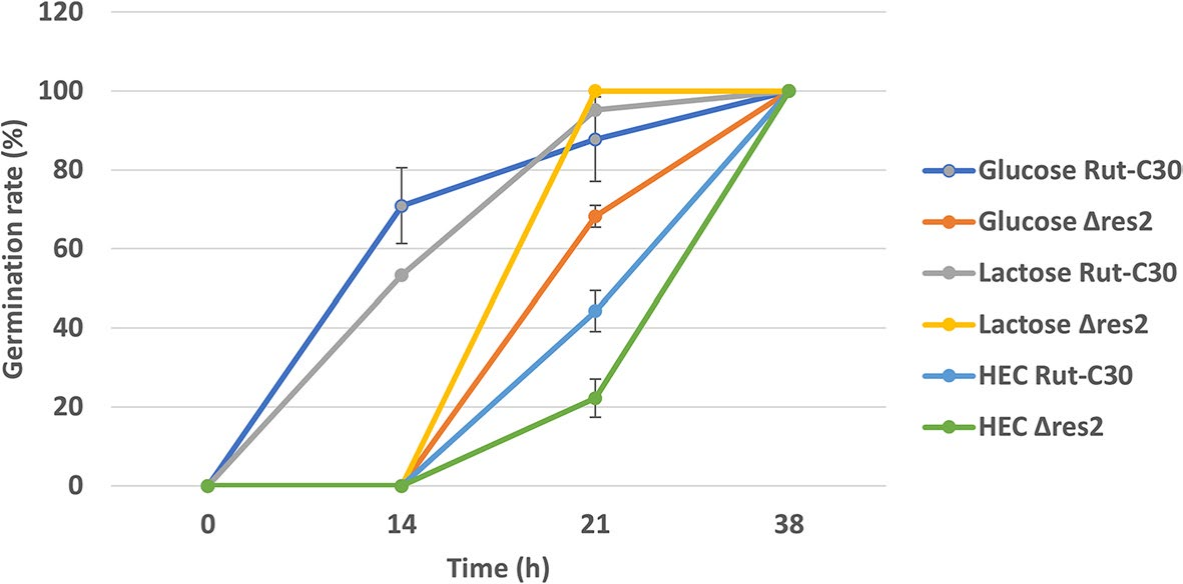
Germination rate of Rut-C30 and Δ*res*2 glucose, lactose and HEC. The error bars indicate the standard deviation of three biological replicates of one representative transformant of Δ*res2*

To investigate if growth was already affected at the germination step, we assessed the germination rate for both Rut-C30 and the Δ*res2* transformant 2 on three media: minimal medium with glucose as a non-inducing condition, and minimal medium containing lactose or soluble hydroxyethyl cellulose (HEC) as inducing substrates. Hyphal growth was detected after 14 h on glucose and lactose for the Rut-C30 strain (Additional file 1 and Fig. 2). Δ*res2* showed slower germination on the three substrates. On HEC, hyphae appeared after 21 h for both strains with a slight delay for the mutant strain (Fig. 2**)**. However, even if the germination rate was not or not much reduced for the Δ*res2* after 21 h on the three substrates, it can clearly be seen on Additional file 1 that the hyphal length of the mutant was much shorter.

### Deletion of *res2* leads to reduced protein production

**Fig. 3:**
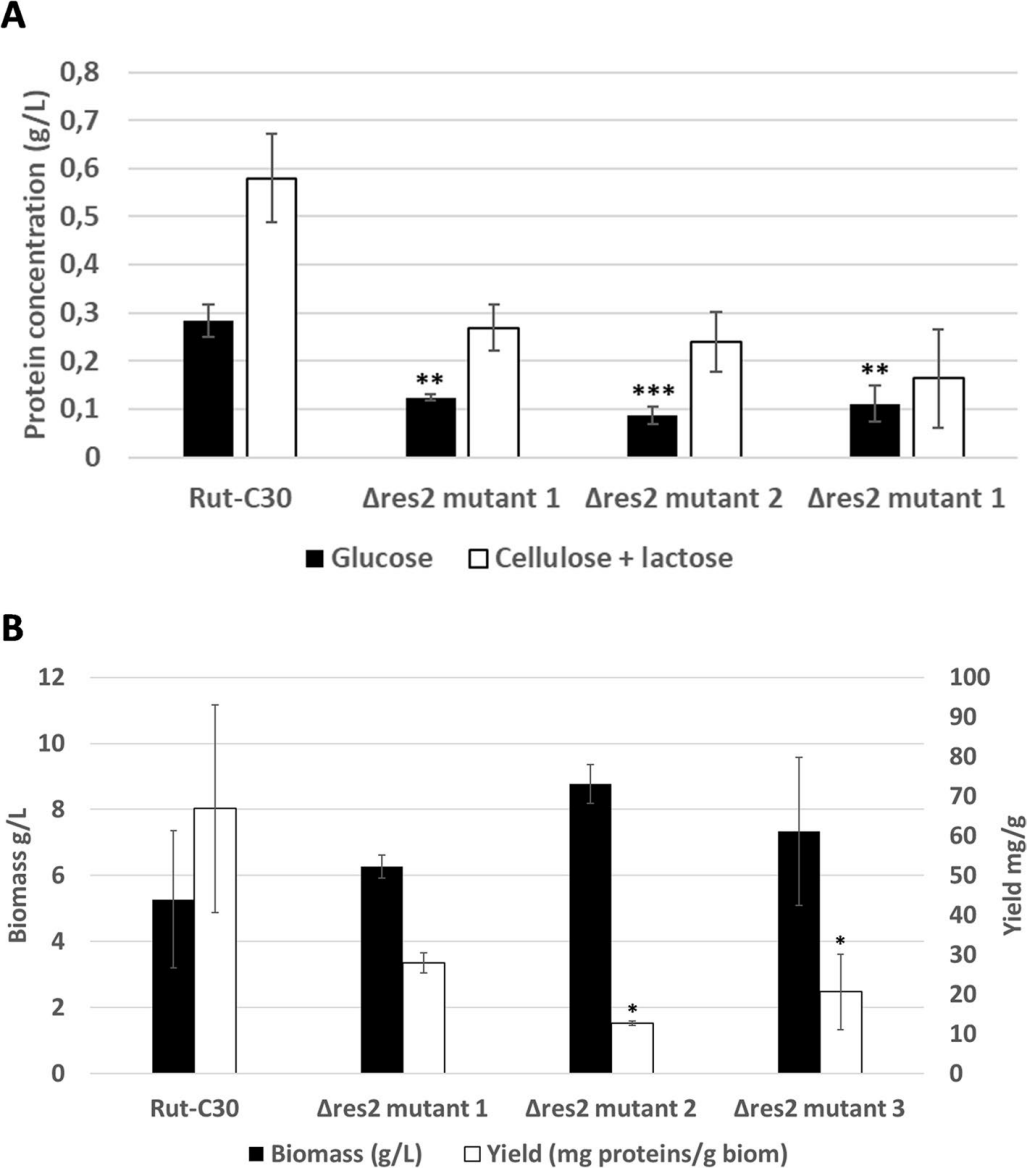
**A** Concentration of secreted proteins of ∆*res2* mutant and its parental strain Rut-C30. **B** Biomass and yield of ∆*res2* and Rut-C30 in glucose cultures. Strains were cultured for seven daysin 25 mL of BTCA medium in the presence of either glucose or cellulose and lactose. All cultures were inoculated with the same number of spores (~2x10^5^ spores). The error bars indicate the standard deviation of three biological replicates for glucose cultures and two biological replicates for cellulose + lactose cultures. Significance of the difference between the Rut-C30 and the mutant strains was calculated with the two-tailed t-test with independent variables (*P < 0.05, **P < 0.01, ***P < 0.001)

Protein secretion of the two strains was quantitatively measured after growing them in shake flasks in the presence of either glucose or cellulose + lactose. As expected, higher protein secretion was observed in the cellulase-inducing condition than with glucose for both strains. However, protein secretion in the three *∆res2* mutants was lower compared to that in the Rut-C30 parental strain (Fig. 3A). The biomass of the mutants and the parental strain were very similar or even slightly higher in the mutants, leading also to a reduced protein production yield in the *∆res2* strains (Fig. 3B). These results are in agreement with those of [19] where protein secretion decreased approximately by 30% in *N. crassa ∆res2* strains grown on 1% w/v crystalline cellulose supporting the hypothesis that the TF Res2 is involved the regulation of protein secretion.

### Impact of secretion stress on gene expression in Rut-C30 and ∆*res2*

**Fig. 4:**
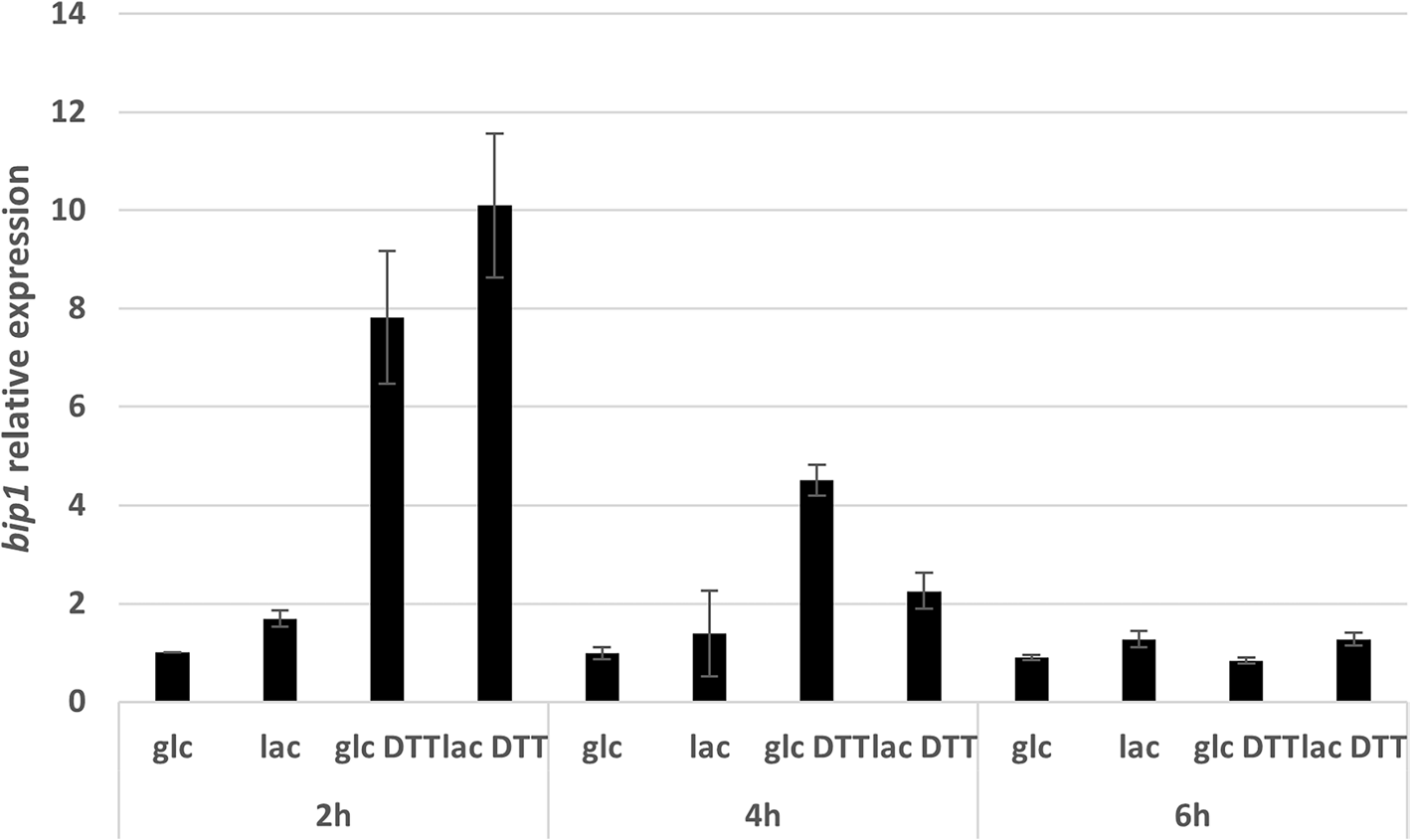
Relative gene expression of the secretion stress biomarker *bip1* in Rut-C30 in fed-batch cultures. RNA samples were taken at three timepoints (2h, 4h, 6h) after the addition of DTT. The relative expression of *bip1* with respect to that on glucose at 2h is shown. The error bars indicate standard deviation of two biological replicates

To determine if RES2 plays a role in the secretion pathway regulation or the secretion stress response in the hyperproducer Rut-C30, a transcriptomic study was conducted in conditions inducing secretion stress. It had previously been demonstrated that growth of Rut-C30 in lactose fed-batch culture [18] and addition of DTT [12] to *T. reesei* culture induce UPR. To choose appropriate stress conditions, preliminary fed-batch cultures on lactose and glucose with or without addition of DTT were conducted and the transcript levels of the UPR marker gene *bip1* determined at different time points by qPCR. Fed-batch fermentation allows to obtain a higher protein production in lactose culture and to control the pH, thus reducing variables that can affect gene regulation and mimicking industrial like conditions. The results showed that induction of cellulase production by lactose feeding and exposure to 10 mM DTT led to an increase in the UPR marker gene *bip1* (Fig. 4). The highest increase of *bip1* transcript levels was detected at 2 h after addition of DTT. In the lactose cultures, there was only a low increase of *bip1* mRNA levels compared to the glucose cultures, indicating a moderate secretion stress. Similarly to *bip1*, transcript levels of the UPR marker genes *pdi1* and *hac1* (spliced version) were also highest 2 h after DTT addition (Additional file 2). In addition, lactose induced the transcription of cellulases as expected, evidenced by an increase of the transcription levels of the *cbh1* gene at all time points. However, the *cbh1* gene was repressed after 4 and 6 h in the glucose culture with DTT, and much less induced in lactose cultures with DTT (Additional file 2). Taking into account these results, we decided to analyze gene expression by RNA-seq 2 h after exposure (D) or not (C) to 10 mM DTT in the reference strain Rut-C30 (R) and one representative transformant of the Δ*res2* mutant (∆r) in glucose (G) and lactose (L) fed-batch cultures, leading to four culture conditions : Glucose Control (GC), Glucose DTT (GD), Lactose Control (LC), Lactose DTT (LD). Transcriptome data were used for pair-wise comparison of cultures within each strain (GD/GC, LD/LC, LC/GC, or LD/GD) and between strains for the same conditions. The results of the fed-batch fermentations (protein concentration and biomass) are presented in Additional file 3.

**Fig. 5:**
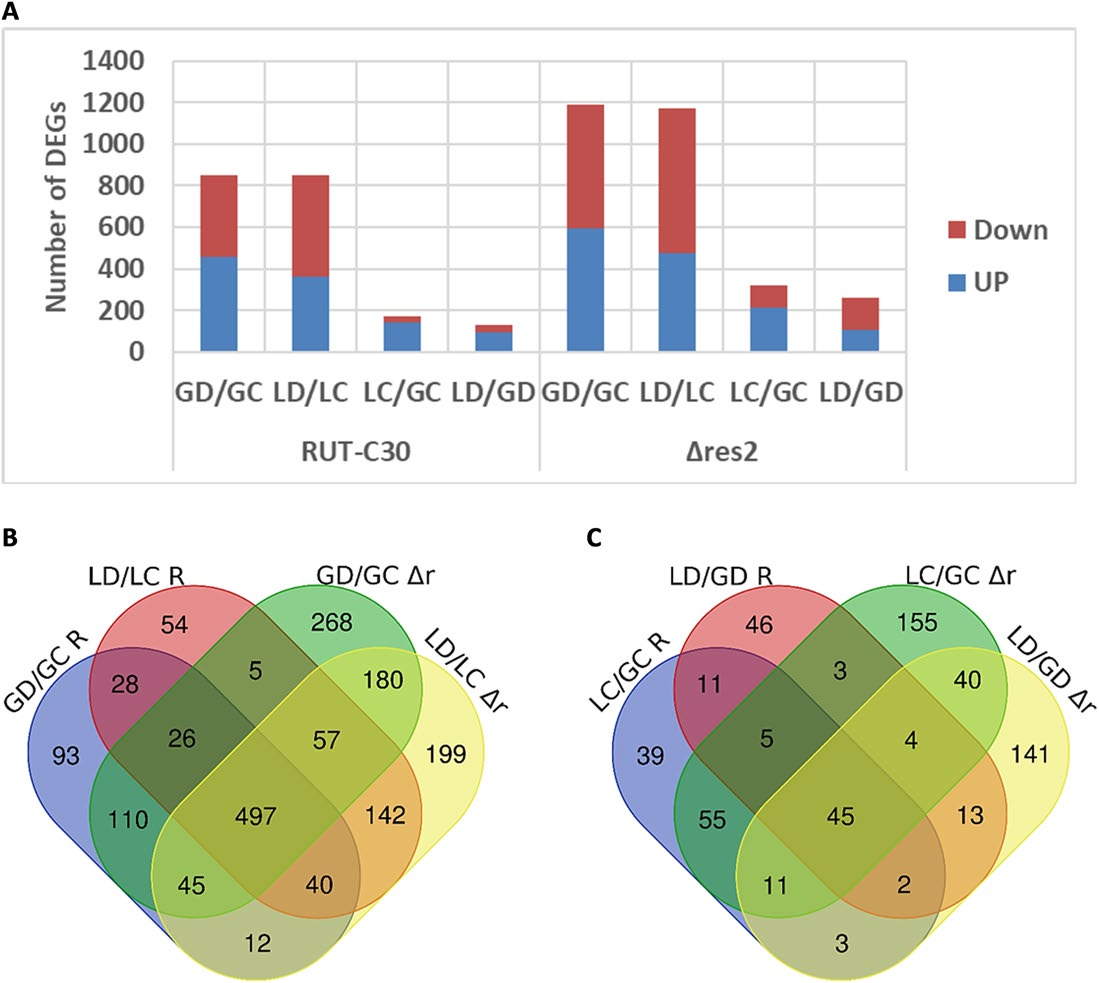
DEGs of Rut-C30 (R) and Δ*res2* (Δr) in two stress conditions. **A** Bar graph displaying the number of up and down-regulated genes in Rut-C30 and Δ*res2* in each condition using a 0.5% false discovery rate cut-off and with an absolute log2 fold change greater than 2. Venn diagrams showing both unique and common DEGs in Rut-C30 and Δ*res2* in **B** the presence vs. absence of DTT and **C** glucose vs. lactose cultures

A total of 1163 and 1664 genes (~ 10% of the genome) were differentially expressed (DE) in at least one of the four comparisons in the Rut-C30 and the Δ*res2* strains, Additional files 4 and 6, respectively. Exposure to 10 mM DTT led to a much higher change in gene expression in Rut-C30: 851 and 849 Differentially Expressed Genes (DEGs) in GD/GC and LD/LC compared to 171 and 129 in lactose-induced stress conditions LC/GC and LD/GD, respectively. Similarly, 1188 and 1172 genes were DE in Δ*res2* in GD/GC and LD/LC compared to 318 and 259 in LC/GC and LD/GD, respectively. Remarkably, in Δ*res2* more genes were differentially expressed genes than in Rut-C30. Whereas approximately as many genes were up – and downregulated due to the addition of DTT, there were much more upregulated genes in the lactose compared to the glucose cultures for both strains. The only exception is the LD/GD of the Δ*res2* strain with more down- than upregulated genes (Fig. 5A).

Out of 1163 DEGs, only 192 (~ 16.5%) were uniquely DE in Rut-C30 while 693 (~ 41.6%) out of 1664 DEGs were uniquely DE in Δ*res2*. 971 DEGs were common between the two strains. 934 genes (~ 56%) of the DEGs in Δ*res2* were common with Rut-C30 in DTT-induced stress conditions (GD/GC and LD/LC) (Fig. 5B). What is intriguing is that only few DEGs (141, ~ 8.5%) were common between the two strains in the lactose-induced stress conditions (LC/GC and LD/GD) (Fig. 5C). Most of them code for CAZymes (e.g. CEL3D), and a smaller number code for transporters (e.g. TrSTR1) or redox regulators (e.g. AOD1). Interestingly, a much higher number of genes, i.e. 336 were differentially regulated in the Δ*res2* strain only, but only 175 were unique to Rut-C30 in the lactose conditions.

### *res2* and Rut-C30 have similar clusters of DE genes

**Fig. 6:**
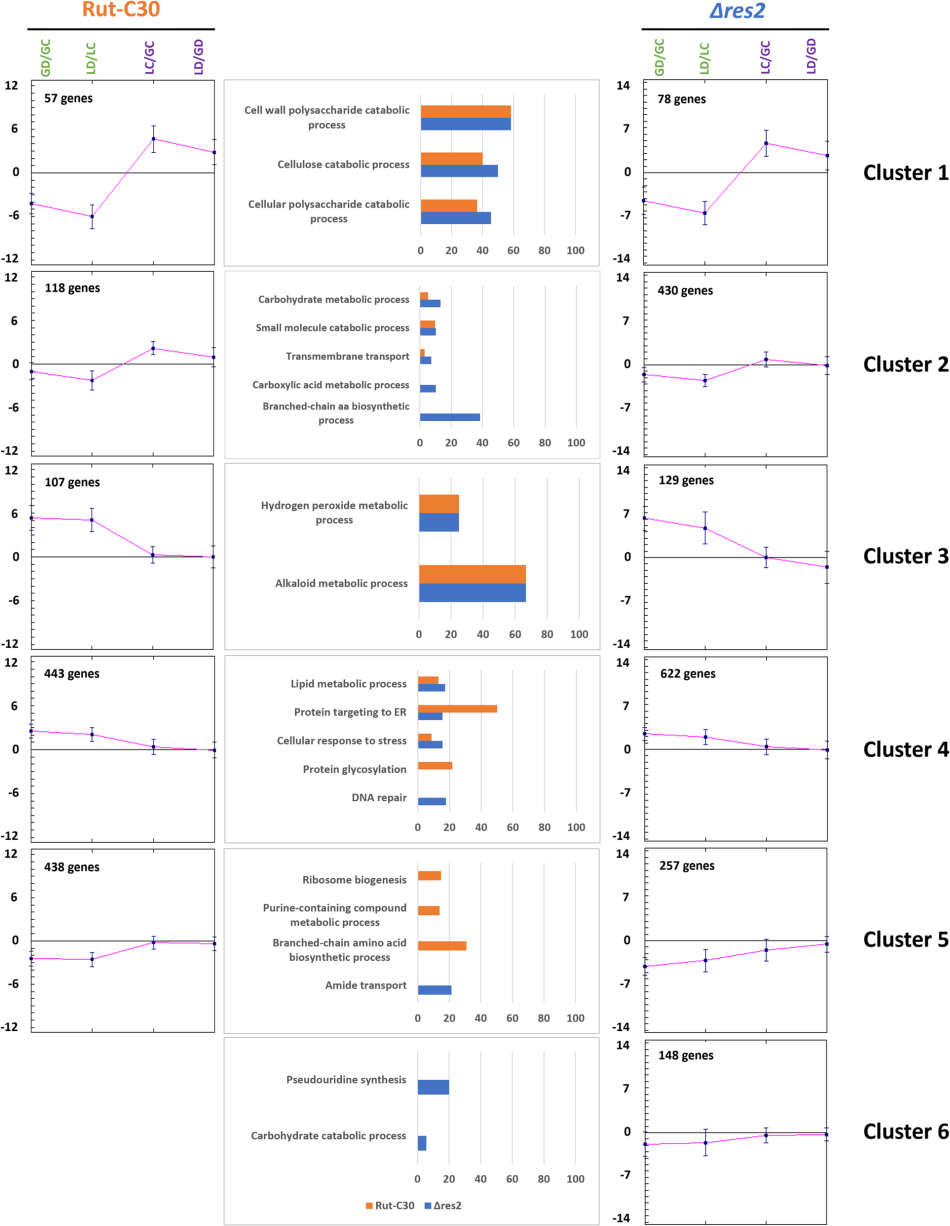
Clustering of the DEGs. The average profile of each cluster is shown for Rut-C30 (on the left) and Δ*res2* (on the right). The histograms in the middle represent the most enriched GO terms expressed as a percentage of genes compared to the number of background genes for each cluster of the two strains based on fungiDB. Only GO terms with a *p*-value < 0.005 are indicated

A clustering analysis of DEGs of both strains was performed. Five different expression profiles were found for Rut-C30 and Δ*res2*, but Δ*res2* had a sixth cluster of unassigned genes (Fig. 6). The first five clusters display very similar profiles.

Gene ontology enrichment analysis (Additional files 5 and 7) revealed that the first cluster was enriched in genes encoding secreted proteins such as cellulases and other lignocellulose-degrading enzymes (e.g. TRIREDRAFT_123989 *cel7a/cbh1*, TRIREDRAFT_74223 *xyn1*, TRIREDRAFT_76672 *cel3a/bgl1*) in both strains. Transcript levels were greatly reduced in the presence of DTT, both with lactose and glucose as carbon source, and significantly increased in the presence of lactose compared to the glucose control culture. Rut-C30 had an additional enriched GO term cell wall polysaccharide metabolic process (e.g. TRIREDRAFT_120229 *xyn3* and TRIREDRAFT_121127 *bxl1*). Thus, the *res2* deletion did not impact lactose induction of genes involved in biomass degradation.

The second cluster contained genes involved in carbohydrate metabolic process for both strains. These genes (e.g. TRIREDRAFT_104797 *bgl3j* and TRIREDRAFT_46816 *cel3d*) showed moderately lower expression levels in the presence of DTT and either no change in expression in the presence of lactose or some increase in transcript levels. In addition, small molecule metabolic process genes (e.g. TRIREDRAFT_110414 *uga1* and TRIREDRAFT_123288 *xki1*) and transmembrane transport (e.g. TRIREDRAFT_104072 *xlt1*) were also enriched. Some GO terms were specifically enriched in Δ*res2* with 430 genes in this cluster, compared to only 118 in Rut-C30: carboxylic acid metabolic process (e.g. TRIREDRAFT_102382 *glo2* and TRIREDRAFT_121449 *his3*) and branched chain amino acid metabolism (e.g. TRIREDRAFT_122868 *hom6* and TRIREDRAFT_51499 *ilv5*) were found to be enriched.

The third cluster containing approximately a hundred genes in both strains was characterized by a high upregulation of genes (e.g. TRIREDRAFT_106245 *cta1* and cytochrome P450-encoding genes) acting against oxidative stress in conditions where DTT was present. An increase in transcript levels of Redox active genes is unsurprising as DTT is a reductant that has a variety of stress effects on fungal cells.

The fourth cluster grouped genes which displayed upregulation in the presence of DTT, but at a lower level than genes in cluster 3. Additionally, they were also slightly upregulated on LC/GC. Genes in this cluster have functions in protein targeting, secretion and lipid metabolism. Also, UPR genes such as TRIREDRAFT_122920 *bip1* and TRIREDRAFT_122415 *pdi1*, as well as genes of the ERAD pathway like TRIREDRAFT_50647 *hrd1* and TRIREDRAFT_47330 *lcl2* fall in this group. In Rut-C30, protein glycosylation related genes were also enriched, whereas the GO terms DNA repair and cellular response to stress were more specifically enriched in the mutant.

Although the fifth cluster grouped genes with similar gene expression profiles, i.e. a moderate downregulation with DTT and in LC/GC, the most enriched GO terms in each strain were different. The three most enriched GO terms in RUT-C30 were ribosome biogenesis (e.g. TRIREDRAFT_104595 *snu13* and TRIREDRAFT_124149 *nhp2*), purine-containing compound metabolic process (e.g. TRIREDRAFT_120568 *eno1* and TRIREDRAFT_47221 *ynk1*) and branched-chain amino acid biosynthetic process, while in Δ*res2*, genes related to amide transport (e.g. TRIREDRAFT_59364 *opt2*) were enriched. For the sixth cluster of unassigned genes in Δ*res2*, no specific enrichment of functions was found, but it contained genes coding for proteins with putative functions in pseudouridine synthesis (e.g. TRIREDRAFT_3671 *gar1* and TRIREDRAFT_44449 *cbf5*) or carbohydrate catabolic process (e.g. TRIREDRAFT_121735 *cel3b* and TRIREDRAFT_55319 *abf2*).

### Function of differentially regulated genes in the Δ*res2* strain

Although clustering did not reveal major differences in global the gene expression patterns between Rut-C30 and the *res2* deletion strain, we looked for individual genes that were differentially regulated in both strains. To potentially identify genes related to the protein secretion pathway, we focused on the fourth cluster grouping many of these genes of which 443 and 622 (~ 5% of the genome) were DE in Rut-C30 and Δ*res2*, respectively. However, genes involved in secretion or secretion stress response such as UPR or ERAD were not differentially expressed in the Δ*res2* strain compared to Rut-C30 under any condition implying that RES2 is not involved in the regulation of the secretion stress response. But several other genes related to the secretion pathway and to other metabolic functions displayed differential expression in the two strains in various clusters and are described in more detail below.

As DTT might impact a lot of cellular functions and lead to a high number of DEGs, we first concentrated on lactose-induced stress conditions (LC/GC or LD/GD). This condition more specifically highlights DEGs that are potentially involved in protein secretion rather than other kinds of biological processes. For example, some MFS (Major facilitator superfamily) and ABC transporters were found to be upregulated in the condition LC/GC in Δ*res2* but not regulated in Rut-C30, such as low-affinity glucose transporter TRIREDRAFT_106556 (*hxt13*), TRIREDRAFT_62747, and TRIREDRAFT_47897 which is involved in the oxidative stress response. One MFS transporter in this cluster is downregulated in the mutant (TRIREDRAFT_58561). On the other hand, TRIREDRAFT_61278 encoding a putative high affinity glucose transporter was upregulated specifically in Rut-C30 in the LD/LC condition (Table 1). Another gene, TRIREDRAFT_124198 coding for a putative secreted protein of unknown function, displayed a Log2 fold change (L2FC) that increased from 1.56 in Rut-C30 to 4.3 in Δ*res2* in the condition LC/GC.

**Table 1:** Significantly differentially regulated genes in only one of the strains in the indicated condition(s)

	Gene ID	Gene name	Putative function	DE in condition	Log2 fold change	Subcellular locilization	Cluster
**Genes DE in Rut-C30 and** ***Δres2***	TRIREDRAFT_50607	*are2*	sterol O-acyltransferase	LC/GC	2.33 (Δ*res2*)	ER	4
	TRIREDRAFT_77547		UDP-glucose:sterol glycosyltransferase	LC/GC LD/GD	-4.38 (Δ*res2*) -2.58 (Δ*res2*)	cytoplasm	4
	TRIREDRAFT_109980		Putative Acyltransferase CST26	LD/LC	2.01 (Rut-C30)	PM, ER	4
	TRIREDRAFT_109234		Putative D-aminopeptidase	GD/GC LD/LC	3.63 (Δ*res2*) 2.15 (Δ*res2*)		4
	TRIREDRAFT_67699		Putative Amino acid transporter	GD/GC	3.09 (Rut-C30)		4
	TRIREDRAFT_106315		Putative Trypsin-like serine proteases domain protein	LD/LC	-2.25 (Rut-C30)		2
	TRIREDRAFT_53961		Secreted Aspartic protease PEP2	LC/GC	-2.23 (Δ*res2*)		5
	TRIREDRAFT_120953		Putative Glycoside hydrolase family 18	LC/GC	3.25 (Δ*res2*)		5
	TRIREDRAFT_65891	*pks2*	Polyketide synthase-6	LC/GC	2.06 (Δ*res2*)		4
***Δres2*** **unique genes**	TRIREDRAFT_45980		Putative protein involved in phospholipid translocation	LC/GC	2.11	PM	4
	TRIREDRAFT_22331	*pldB*	Putative phospholipase D active site protein	LC/GC	2.90		4
	TRIREDRAFT_120125		Putative Lipase/serine esterase	GD/GC	2.34	cytoplasm	4
	TRIREDRAFT_55627	*ept1*	Putative Ethanolaminephosphotransferase	GD/GC	2.06	ER, Golgi	4
	TRIREDRAFT_81972	*Section 14*	Putative Phosphatidylinositol transfer protein similar to Sect. 14 involved in intracellular transport and UPR	GD/GC	2.06	cytoplasm	4
	TRIREDRAFT_72788		Putative Glycosyltransferase family 31	GD/GC	2.05	ER	4
	TRIREDRAFT_123718		Putative Amino acid transporter	GD/GC	2.29		4
	TRIREDRAFT_77283		Putative Glycosyltransferase family 2	GD/GC LD/LC	2.31 2.53	ER or PM	4
	TRIREDRAFT_120923		Putative Glycosyltransferase family 32	GD/GC LD/LC	8.43 2.98	ER	3
	TRIREDRAFT_121486		Putative Amino acid transporter	GD/GC	7.74		3
	TRIREDRAFT_21960	*ple*	Putative Phospholipase E	LC/GC	2.97	PM	6
	TRIREDRAFT_73248		Putative Glycoside hydrolase family 55	LC/GC	-2.17		6
	TRIREDRAFT_121746		Putative Glycoside hydrolase family 55	GD/GC	-2.20		6
	TRIREDRAFT_106556	*hxt13*	hexose transporter	GD/GC LD/LC LC/GC	5.08 3.14 6.67		4
	TRIREDRAFT_62747		MFS membrane transporter	LC/GC	3.16		4
	TRIREDRAFT_47897	*snq2*	ABC transporter involved in oxidative stress response	GD/GC LC/GC	2.08 3.17		4
	TRIREDRAFT_58561	*tpo4*	MFS permease	LC/GC	-2.14		4
	TRIREDRAFT_46285	*hsp30*	Heat shock protein 30	LC/GC	3.24		4
	TRIREDRAFT_77770		Zinc-binding alcohol dehydrogenase	GD/GC LC/GC	2.75 2.18		4
	TRIREDRAFT_27948	*csg1*	G-protein-coupled receptor involved in cellulose sensing CSG1	GD/GC LC/GC	2.81 2.16		4
	TRIREDRAFT_81049	*erg3*	Ergosterol 3	LD/LC	-2.06		2
	TRIREDRAFT_36822		Putative Glutathione S-transferase	LC/GC	-4.46		5
**Rut-C30 unique genes**	TRIREDRAFT_122992		Putative GT31 ß-Glycosyltransferase	LC/GC	3.14	ER	2
	TRIREDRAFT_105784		DUF636 family protein, putative transcription factor	GD/GC	2.80		4
	TRIREDRAFT_66687		Putative Glycosyltransferase family 17	GD/GC	2.01	Golgi	4
	TRIREDRAFT_64925		Putative Glycosyltransferase family 32	GD/GC	-2.08	ER	2
	TRIREDRAFT_120873		Putative Glycoside hydrolase family 71	LC/GC	-2.66		5
	TRIREDRAFT_61278		High affinity glucose transporter	LD/LC	3.68		4
	TRIREDRAFT_68941		Putative Glutathione S-transferase	GD/GC	3.19		4

*PM* Plasma membrane

Other upregulated genes in LC/GC in Δ*res2* but not DE in Rut-C30 include TRIREDRAFT_46285 encoding the chaperon HSP30, the putative alcohol dehydrogenase involved in redox reactions TRIREDRAFT_77770 and *pks2* (TRIREDRAFT_65891) having roles in secretion and secretion metabolism. Also, a G-protein-coupled receptor involved in cellulose sensing *csg1* (TRIREDRAFT_27948) is upregulated in GD/GC and LC/GC in in Δ*res2* only and falls in cluster 4.

Interestingly, several lipid metabolism genes of this cluster were specifically DE in the lactose culture in the mutant strain, such as phospholipase D (*pldB*, TRIREDRAFT_22331) and TRIREDRAFT_45980, a gene encoding a protein putatively involved in phospholipid translocation. They are both upregulated in the in Δ*res2* strain in this condition (Table 1). The *ple* gene (TRIREDRAFT_21960) encoding phospholipase E showed a similar upregulation in LC/GC but was grouped in the unassigned genes cluster of Δ*res2*. On the other hand, the acyltransferase CST26 gene (TRIREDRAFT_109980) was induced in Rut-C30 only in the LD/LC condition. This *Candida albicans* orthologue encodes a transferase involved in phospholipid synthesis and its lack of induction suggests that its expression could be regulated by RES2 (fungiDB).

An important component of fungal membranes is ergosterol. But only two genes of the ergosterol pathway were found to be differentially regulated in the Δ*res2* strain compared to Rut-C30: the *are2* gene encoding a sterol-O-acyltransferase (TRIREDRAFT_50607) and erg3, a putative C-14 sterol reductase (TRIREDRAFT_81049). The former was specifically induced in LC/GC whereas the latter was repressed in the presence of both DTT and lactose (LD/LC) in the mutant strain. Therefore, biosynthesis of ergosterol did not seem to be significantly affected by the lack of RES2.

In yeast and filamentous fungi, UPR is linked to cell wall integrity [21] and in yeast, the two pathways are coordinately regulated. In Rut-C30 and the Δ*res2* mutant, UPR is induced with DTT and cellulase induction by lactose. Even if UPR related genes were not DE in the two strains, we verified if the expression of genes coding for putative cell wall modifying enzymes were differentially affected. Indeed, a chitinase (TRIREDRAFT_120953), two β-1,3 glucanases belonging to family GH55 (TRIREDRAFT_73248 and TRIREDRAFT_ 121746) and a GH71 α-1,3 glucanase were differentially regulated in the two strains in either of the two secretion stress conditions. The GH18 gene was induced in LC/GC in the Δ*res2* strain only whereas both GH55 genes were repressed in the same strain in the presence of either lactose or DTT. GH71 gene is downregulated in LC/GC, but only in Rut-C30 (Table 1). All four proteins are predicted to be secreted or cell wall located.

We also analyzed the effect of the *res2* deletion on media containing the redox stress agent DTT in more detail. Again, genes encoding proteins involved in the translocation or intracellular transport of phospholipids were found to be upregulated uniquely in the Δ*res2* mutant (*ept1*, TRIREDRAFT_55627 and *Sec14*, TRIREDRAFT_8192). These results suggest that the deletion of *res2* impacts the expression of genes involved in the ER membrane synthesis or homeostasis, which is particularly important in conditions of secretion stress.

Another functional group of genes for which the expression was found to be impacted by the deletion of *res2* encode glycosyltransferases (GT). Among other functions, these enzymes are catalyzing several protein glycosylation steps during maturation in the ER and Golgi. Three GT (TRIREDRAFT_72788, TRIREDRAFT_77283 and TRIREDRAFT_120923) belonging to CAZy families GT2, GT31 and GT32 were upregulated in the presence of DTT, but in the Δ*res2* mutant only. Three other GT (TRIREDRAFT_66687, TRIREDRAFT_64925 and TRIREDRAFT_122992) were upregulated with DTT uniquely in Rut-C30. It is noteworthy that all the mentioned GT whose genes were DE are predicted, by localization prediction online tools, to be located in the ER or Golgi apparatus. Even if it is not possible at this stage to evaluate the real impact on glycosyl side chains of secreted proteins, glycosylation is probably altered in the Δ*res2* mutant in the presence of DTT which could interfere with normal secretion and/or activity of secreted enzymes.

These results suggest that RES2 is, probably among other functions, somehow involved in the regulation of protein synthesis and protein fate and of pathways related to secretion. It is not known if RES2 acts directly on the regulated genes or if its action involves other factors. Therefore, we analyzed the data for differentially regulated TFs. In Rut-C30, only two genes encoding putative transcriptional regulators were DE compared to the Δ*res2* strain, one up- and the other downregulated (Table 2). In contrast, in the latter, six genes were specifically DE in the deletion strain and four of them were downregulated in the presence of DTT. TRIREDRAFT_109538 belonging to cluster 4 was upregulated in the GD/GC condition whereas the sixth one, TRIREDRAFT_12107, a homeodomain-containing protein, was upregulated in the lactose culture in the mutant only. Interestingly, the ortholog of this gene in *Penicillium oxalicum* was found to be involved in cellulase and xylanase production [22, 23]. Finally, the VIB1 TF (TRIREDRAFT_54675) which belongs to cluster 2 was downregulated in both strains in the presence of DTT (GD/GC and LD/LC) but upregulated by Log2fold change factor of 1.85 in the presence of lactose in the Δ*res2* strain only. This points to an eventual interaction between the two transcription factors in cellulase inducing conditions.

**Table 2:** Genes encoding putative transcriptional regulators differentially regulated in only one strain in the indicated condition

	Gene ID	Gene	DE in condition	Log2fold change	Cluster
**Rut-C30 unique genes**	TRIREDRAFT_121757	C2H2 transcription factor tfIIIA	LD/LC	-2.12	5
	TRIREDRAFT_105784	DUF636 domain-containing protein	GD/GC	2.8	4
***Δres2*** **unique genes**	TRIREDRAFT_109538	Putative BZIP transcriptional regulator	GD/GC	4.13	4
	TRIREDRAFT_121074	homeobox transcriptional regulator *hom3*	LC/GC	2.10	2
	TRIREDRAFT_55272	Putative Zn(2)Cys6 transcription factor	GD/GC	-5.20	5
	TRIREDRAFT_76590	Zn2Cys6 transcriptional regulator *pro1/rosA/adv1*	GD/GC	-2.95	Unassigned
	TRIREDRAFT_105255	Zn2Cys6 transcriptional regulator	GD/GC	-2.16	Unassigned
	TRIREDRAFT_121682	Zn2Cys6 transcriptional regulator	GD/GC	-2.62	Unassigned

## Discussion

In this study, the role of the TF RES2 in secretion and the secretion stress response in *T. reesei* Rut-C30 was investigated. We could show that deletion of the *res2* gene resulted in reduced radial growth on glucose and lactose and slower germination as well as in decreased productivity in both non-inducing and cellulase secretion inducing conditions. The phenotype observed resembles the one obtained in the *N. crassa res2* deletion mutant but is not identical. In *N. crassa*, growth of the Δ*res2* mutants on minimal media was indistinguishable from the WT strain on minimal media and reduced only at concentrations > 5 mM DTT [19]. In our case, we could not find a negative effect of DTT on the mutant up to 5 mM DTT, the highest concentration tested. Addition of DTT even seemed to promote growth in the mutant strain, an unexpected result. However, growth of the deletion strain was clearly reduced in the absence of DTT on all carbon sources compared to Rut-C30.

In this context, it is important to mention that despite the visibly slower growth in solid cultures, biomass production in liquid cultures was not reduced in the mutant and the lower protein secretion could thus not be explained by a reduced growth. The difference in extracellular protein concentration was therefore due to a less efficient secretion, both in non-inducing and inducing conditions.

When comparing *T. reesei* to *N. crassa*, one must keep in mind that regulation of cellulase gene expression implies different transcription factors in these species. Whereas in *N. crassa*, CLR-1 and CLR-2 are essential for cellulase gene activation [24], XYR1 and ACE3 are the main regulators in *T. reesei* [25, 26]. Indeed, no orthologous genes of *clr-1* and *clr-2* are present in the latter. Another important fact is that the present study was done on the hyperproducing strain Rut-C30 in which cellulase genes are highly inducible and where the role of RES2 might be altered compared to the wild type strain QM6a. However, it is also worth mentioning that there are no mutations in the sequence of *res2* gene in the hyperproducing strain Rut-C30 compared to that in the wild-type strain QM6a and that the expression of this gene is similar in the QM6a strain and the Rut-C30 both in glucose- and lactose-containing media (F. Bidard-Michelot, unpublished results).

In accordance with previous studies, we observed downregulation of transcription of many secreted proteins (mainly cellulases) in DTT stress conditions. This can be explained by repression under secretion stress (RESS), since induction of this stress response mechanism by DTT in Rut-C30 was already evidenced before [12]. The reduction of cellulase gene expression by DTT is the same in Rut-C30 and Δ*res2*, which indicates that RES2 is not involved in the regulation of this mechanism. It does not seem to play a significant role in triggering the UPR or ERAD response either, as *pdi1* and *bip1* as well as most ERAD-related genes, such as *hrd1*, *hrd3*, *lcl2*, and *cpr1* were not DE between the two strains. This is in contrast to the results obtained in *N. crassa*, where RES2 was proposed to be involved in secretion and the secretion stress response [19]. Although the *res2* gene was moderately upregulated in *T. reesei* Rut-C30 in the presence of DTT, similarly to *N. crassa* (Log2 fold change 1,38 and 1,6 respectively), the role of this transcriptional regulator under these stress conditions is apparently different *N. crassa* and in the hypersecreting *T. reesei* strain.

Most oxidoreductases, catalases, dehydrogenases and cytochrome-P450 were upregulated only in DTT conditions. This is expected due to the higher stress induction by the reducing effect of DTT. However, several redox regulators (e.g. TRIREDRAFT_80659 and TRIREDRAFT_77770) were upregulated in Δ*res2* uniquely implying that RES2 plays a role in maintaining a stress-free cellular environment in Rut-C30, and its deletion evokes the release of reactive oxygen species.

As in *N. crassa*, the *res2* deletion was found to decrease secretion of cellulolytic enzymes. However, whereas hemicellulase and cellulase transcription was downregulated in Avicel cultures of the *N. crassa* Δ*res2* strain, the mRNA levels of these genes in the *T. reesei* Δ*res2* were identical to those in the Rut-C30 strain upon induction by lactose. This indicates that RES2 does not contribute significantly to the transcriptional induction of cellulase genes by lactose in Rut-C30. In fact, in this hyper producing strain, the gene of the major transcriptional regulator of lignocellulase genes, XYR-1 is highly expressed, and the high induction mediated by this TF might override more subtle regulations by other TFs such as RES2.

Despite the induction of cellulase encoding genes by lactose in the deletion strain, protein secretion was lower in this condition compared to the parental strain. There are different explanations for this observation. First, it could be the result of a regulation at the translational level. Even if inhibition of translation does not play an important role in ER stress in *Trichoderma* [12], the observed impact of the *res2* deletion on the amino acid biosynthesis pathway could slow down protein elongation. The decrease could also be related to modification steps at the post-translational level such as folding and maturation of the polypeptides in the ER and the secretory pathway. Among the genes specifically DE in the Δ*res2* strain in the presence of lactose we could identify genes involved in phospholipid metabolism (e.g. phospholipase E), a chaperone (TRIREDRAFT_46285), both upregulated, but also genes putatively involved in the modification of the protein glycans (TRIREDRAFT_73248, TRIREDRAFT_77547, both downregulated). It is therefore possible that changes in the ER membrane or the protein glycan side chains lead to less efficient protein secretion. Alternatively, enhanced protein degradation in the extracellular space might also explain the decreased protein concentration. TRIREDRAFT_82623, a putative secreted subtilisin, was specifically induced in the mutant in the presence of lactose, even if the *p*-value of the LC/GC condition was above 0.005 (0.03). As some lipid metabolism genes were regulated, it is possible that RES2 contributes to the highly developed ER structure in Rut-C30. Analysis of the ER structure of the Δ*res2* strain by electron microscopy could verify this hypothesis.

Approximately 5% (459) of the genome of *T. reesei* encode proteins having transport functions (*T. reesei* transporters annotated in the genome portal http://genome.jgi-psf.org/Trire2/Trire2.home.html). Among these genes, there are around 50–100 sugar transporters of which the majority are uncharacterized [27, 28]. Concerning transporter-coding genes that were DE uniquely in Δ*res2* in this study, many of them belong to the MFS superfamily that comprises 16 different families with 89 subfamilies [29] of which each can transport essential nutrients and ions [30] and excrete end products of metabolism [31]. The MFS transporters STR1, CRT1 and STP1 can induce the expression of CAZymes as demonstrated by previous studies [32–34]. CRT1 was demonstrated to have a direct regulatory role on cellulase gene expression, independently of its transporting activity [32]. In our study, the *stp1* gene was not DE in any condition, while *str1* and *crt1* were induced in the presence of lactose but downregulated in the presence of DTT. These lower transcript levels could also have contributed to the downregulation of cellulase gene transcripts in the presence of DTT, in addition to the RESS mechanism. In the Δ*res2* strain, the functional category “transmembrane transport” of uniquely DE genes was enriched (Table 3), and many sugar and amino acid transporters, especially those belonging to cluster 3, were upregulated in GD/GC in this strain only. Others were downregulated in the presence of DTT. The downregulation of transporters in the presence of stress signals was already reported before [35]. As transporters must often be addressed to the plasma membrane, they also pass the ER/Golgi pathway, and it is not surprising that their regulation follows those of secreted proteins.

**Table 3:** Enriched functions of uniquely DE genes in Rut-C30 and Δ*res2*

Rut-C30	Δ*res2*
Ribosome biogenesis and rRNA processing Amine metabolism Nucleotide metabolic process Tryptophane and indole metabolic process Carbohydrate derived metabolic process Amino acid metabolic process	Transmembrane Transport Lipid metabolic Process Valine metabolic Process

We could show that the *res2* deletion led to differential expression of several TFs compared to Rut-C30. All but two of them were differentially regulated (up- or down) in response to DTT in the medium, pointing to a role in the stress response of the fungus to this toxic compound, rather than in protein secretion.

One of them is the cross-pathway control encoding gene *cpc1* which was found to be induced with DTT in both Rut-C30 and Δ*res2* mutant (log2 fold change of 3.3 and 2.6, respectively). This was already observed in other fungi [19, 36] as well as in the work of [13] where also many potential target genes involved in amino acid biosynthesis were induced by DTT. In our study, amino acid synthesis genes were found to be DE in both strains, but with some differences: tryptophane biosynthesis genes were enriched uniquely in Rut-C30 and were upregulated, while valine biosynthesis genes were enriched in the mutant, but downregulated (Table 3). A hypothesis is that RES2 interacts with CPC1 to finetune the expression of AA metabolic genes. But as *cpc1* transcript levels were identical in the mutant and Rut-C30, RES2 does apparently not control the expression of *cpc1*. Yet, it would be interesting to investigate if CPC1 has a role in the regulation of the *res2* gene.

## Conclusions

In an effort to decipher the regulatory mechanism of protein secretion in the hyperproducer *T. reesei* Rut-C30, the role of the RES2 transcription factor was investigated. We could show that the deletion of *res2* impacts fungal growth, germination, and protein secretion. Transcriptomic analysis revealed that CAZyme gene expression was not dependent on the action of RES2 which had no regulatory role on most elements of the stress response mechanisms UPR, ERAD and RESS either. The results are therefore rather in favor of an indirect action of RES2 on protein secretion by modulating the expression of transporters, lipid metabolism and protein modification genes. A major regulatory role of RES2 in amino acid synthesis was also evidenced. As a consequence, it would be interesting to dedicate future studies to the elucidation of a potential interplay between RES2 with other transcription factors such as CPC1. A comparison with the regulatory network involving RES2 in the wild-type strain QM6a could also be interesting and deliver some clues for understanding the hypersecreting phenotype of Rut-C30.

## Methods

### Strains and media

Rut-C30 (ATCC 56,765) strain was used in this study, and the Δ*res2* mutant was derived from this strain. Butanetetracarboxylic acid (BTCA) medium, which was used to culture Δ*res2* and Rut-C30 for protein measurement experiments, was composed of 5.6 g/l (NH_4_)_2_SO_4_, 4.4 g/l K_2_HPO_4_, 0.3 g/l MgSO4.7H_2_O, 0.15 g/l CaCl_2_.2H_2_O, 5.85 g/l BTCA, 3 g/l KOH crystals, 1.5 g/l cornsteep (SOLULYS®) and oligo-elements (30 mg/l FeSO_4_.H_2_0, 9 mg/l Co(NO_3_)_2_.6H_2_0, 6.4 mg/l MnSO_4_.H_2_0, 8.4 mg/l ZnSO_4_.7H_2_0, 3 mg/l CuSO_4_.5H_2_0, 0.4 mg/l H_3_BO_3_, and 1 mg/l MoNa_2_O_4_.2H_2_O). 2% of glucose, lactose or 1% lactose and 1% TechnoCel BH200 (200 μm fibers, TECHNOCEL®) were added as carbon source. Minimal solid medium for growth kinetics measurement was composed of 5 g/l KH_2_PO_4_, 5 g/l (NH_4_)SO_4_, 11.7 g/l Tri-Sodium Citrate, 20 g/l (2%) of carbon source (glucose or lactose), 20 g/l agar, the pH was adjusted to 6. To examine the germination of the mutant strain Δ*res2* and its parental strain Rut-C30, 2 × 10^5^ spores were diluted 200x and spread on plates containing 2% carbon source and solid minimal media. Hyphal growth and branching were observed using microscope ZEISS Imager.M2.

### Fed-flask experiments

Fed-flask experiments were performed in an Infors incubator and using peristaltic pumps (Dasgip MP8) as described by [37] with slight modifications. 250 ml Erlenmeyer flasks containing 60 ml of complete saline medium (1 ml/l H_3_PO_4_ 85%, 2.8 g/l (NH_4_)_2_SO_4_, 300 mg/l MgSO_4_,7H_2_O, 150 mg/l CaCl_2_,2H_2_O, 8 g/l dipotassium phthalate, 1.5 g/l cornsteep, 30 mg/l FeSO_4_,7H_2_O, 6 mg/l MnSO_4_, H_2_O 8 mg/l ZnSO_4_,7H_2_O, 9 mg/l CoNO_3_,6 H_2_O, 1 mg/l H_3_BO_3_, pH = 5.4) supplemented with 12.5 g/l glucose, were inoculated with 2 × 10^5^ spores /ml and cultured at 30 °C with 200 rpm. After a batch phase of two days and verification of glucose consumption (< 0.3 g/l) with a Glucose Analyzer 2 glucostat (Beckman) the fed-batch fermentation was initiated by feeding with a lactose or a glucose solution (55 g/l) at a constant rate of 0.3 ml/h. At 40 h of fed-batch which led to protein secretion in the lactose fed-batch, two replicates of each condition were treated with DTT 10 mM for two hours, while the other two replicates were non-treated. Samples for protein measurements were taken at 0, 20 and 42 h and samples for RNA extraction at 42 h after onset of the fed-batch phase (Suppl. Figure 3).

### RNA sample preparation

8 ml samples were filtered and immediately frozen in liquid nitrogen before being stored at -80 ° C. Frozen mycelia were transferred into a tube of Lysis matrix C (MP Bio) containing 700 µl of RLT lysis buffer (Qiagen, RNeasy Mini Kit (50)) and 7 µl of β-mercaptoethanol. The samples were lysed twice by a Fastprep homogenizer for 40 s at 6 m/s with 5 min resting of samples on ice between each lysis. Then they were transferred to QIAshredder columns, and RNA was extracted as indicated in the instructions of the manufacturer (Qiagen, RNeasy Mini Kit (50)). The RNA concentration was measured with Qubit 2.0, and the quality of the extracted RNAs was evaluated on a Bioanalyzer (Agilent) and agarose gel electrophoresis.

### Measurement of relative gene expression by RT-qPCR

Total extracted RNA was reverse transcribed into complementary DNA (cDNA) using iScript™ cDNA synthesis kit (Bio-Rad, Hercules, USA) and random primers following the instructions of the manufacturer.

To measure relative expression of the secretion stress biomarkers *bip1*, *pdi1* and *hac1*, as well as the cellobiohydrolase gene *cbh1*, qPCRs were done using the iQSYBR Green Supermix (Bio-Rad) and 1 µl of five-fold diluted cDNA in a total volume of 20 µl. The sequence of the primers used are indicated in Additional file 8. Normalization was based on two reference genes *sar1* (Y08636.1) and *glk1* (DQ068384.1) which code for SAR/ARF type small GTPase and glucokinase, respectively [38], and the relative transcript level of each gene was calculated with the Pfaffl method $\frac{\left(\text{E}\text{G}\text{O}\text{I}\right)^{\Delta }\text{C}\text{t} \text{G}\text{O}\text{I}}{\text{G}\text{e}\text{o}\text{M}\text{e}\text{a}\text{n}\left[\right(\text{E}\text{R}\text{E}\text{F})^{\Delta }\text{C}\text{t} \text{R}\text{E}\text{F}}$ [39].

### RNA-seq library preparation and bioinformatic analyses

Library preparation and Illumina sequencing were performed at the Ecole normale supérieure Genomique core facility (ENS Paris, France). Messenger (polyA+) RNAs were purified from 1 µg of total RNA using oligo(dT). Libraries were prepared using the strand specific RNA-Seq library preparation Stranded mRNA Prep kit (Illumina). A 118 bp single read sequencing was performed on a NextSeq 2000 device (Illumina). A mean of 42 ± 6 million sequences passing the Illumina quality filter reads was obtained for each of the sixteen samples (including a biological duplicate for each of the eight samples). Bioinformatic analyses were performed using the Eoulsan (version 2.5) pipeline [40], including read filtering, mapping, alignment filtering, read quantification, normalization and differential analysis: Before mapping, poly N read tails were trimmed, reads ≤ 40 bases were removed, and reads with quality mean ≤ 30 were discarded. Reads were then aligned to the *T. reesei* QM6a genome (JGI version) using STAR (version 2.7.8a) [41]. Alignments from reads matching more than once on the reference genome were removed using htsjdk 1.118 [42]. To compute gene expression, a custom *T. reesei* QM6a annotation file was used. All overlapping regions between alignments and referenced CDS were counted using HTSeq-count 0.5.3 [43]. The sample counts were normalized using DESeq2 1.8.1 [44]. Statistical treatments and differential analyses were also performed with DESeq2 1.8.1. Log2 fold changes were calculated after count normalization, and genes with a mean of at least 100 reads in the respective conditions and displaying a log2fold change > |2| and *p*-value < 0,005 were considered to be differentially expressed.

RNAseq expression data and raw fastq files were deposited in the GEO repository (accession number GSE233738). After filtering DEGs from a total of 10,008 genes, clustering of the DEGs was done using MeV (Multiexperiment Viewer). Gene ontology (GO) of each cluster was obtained using the biological process gene ontology enrichment analysis tool in the fungidb database (https://fungidb.org). Venn diagrams were drawn with the help of an online bioinformatics tool [45]. Intracellular localization was predicted using SignalP − 6.0 [46] and Euk-mPLoc 2.0 [47].

### Construction of the deletion strain

Gene deletions were performed using CRISPR-Cas9. A mix of guide RNA, Cas9 protein forming the ribonucleoprotein complex (RNP) as well as the donor DNA was set up. The Cas9 protein used was purchased from the NEB group (EnGen® Spy Cas9 NLS). Guide RNAs were designed using chopchop online tool [48] and Geneious® Prime bioinformatics software [49]. The deletion cassettes containing the resistance gene hygromycin *hph* flanked by 200 bp homologous to regions on either side of the locus of the target genes were synthesized by Twist Bioscience and PCR amplified. The sequence of gRNAs and Primers can be found in Additional file 8. 10 µL RNP complex containing 1 µl Cas9 (20 µM), 6 µl guide RNA (30 µM), 2 µl of H_2_O DEPC, and 1 µl of 10x Cas9 buffer (NEBuffer™ 3.1) was incubated 10 min at room temperature, then mixed with 50 µl of protoplasts at a concentration of 2.10^8^ ml^−1^ and 5 µg donor DNA (deletion cassette) and incubated for another ten minutes at room temperature. 1 ml of 60% PEG 4000 solution was added and after gentle stirring at room temperature for 20 min, 900 µl of CTS50 (0.4 M saccharose, 0.1 M Tris HCl, 50 mM CaCl_2_, pH 7.5) were added. The transformation reaction was mixed with 40 ml of liquid PDA medium supplemented with 50 µg ml^−1^ hygromycin and 0.8 M of sucrose and poured into five Petri dishes with selective medium containing 50 µg ml^−1^ hygromycin. Single transformants were purified in three successive steps as described by [50].

### Genetic validation of Δ*res2* deletion

DNA was extracted from mycelia after culturing the strains on PD medium for two to four days. DNA extraction was performed using the Nucleospin Soil Genomic DNA kit (Macherey Nagel) and following the soil protocol provided by the manufacturer. PCR was conducted using the Q5 High-Fidelity DNA Polymerase (NEB) and the primers indicated in Additional file 8. 1 ng – 1 µg genomic DNA was used for a 50 µl reaction. In addition to PCRs, the PCR amplicons were sequenced to verify that there were no mutations in the integrated cassette.

To verify the absence of off-targets in Δ*res2* transformants, qPCRs were done on the gDNA of Δ*res2* and two reference strains, each containing two hygromycin cassettes in its genome. Primers targeting the hygromycin resistance gene and the reference gene *bgl2* are indicated in Additional file 8. The copy number of deletion cassettes integrated into the genome of T. reesei was calculated with the Livak method (2^−ΔΔCT^) [51].

### Protein concentration measurement

Protein concentrations of culture supernatants were determined using the Quick Start Bradford protein assay kit (Bio-Rad) and bovine serum albumin (BSA) as a standard. The assay was done on a Freedom Evo robot (TECAN).

### Biomass measurement

To measure the biomass, the mycelium was recovered by filtering 6 mL of the liquid culture on a cellulose filter which has been previously placed at 105 °C for 24 h. After drying the filter with the mycelium at 105 °C for 24 h, the filter was weighed again, and the biomass dry weight was calculated by subtracting the tare.

### Supplementary Information

**Additional file 1.** Hyphal growth of Rut-C30 and Δ*res2* grown on different substrates. After 21h, almost all hyphae started branching in glucose and lactose. Germination was slower on lactose and even slower in HEC.

**Additional file 2. **Relative gene expression of the secretion stress biomarkers *pdi1* and *hac1, *and the cellobiohydrolase gene *cbh1* in Rut-C30 in fed-batch cultures. RNA samples were taken at three timepoints (2h, 4h, 6h) after the addition of DTT. The relative expression is shown with respect to that on glucose at 2h. The error bars indicate standard deviation of two biological replicates.

**Additional file 3.** Protein and biomass profiles of fed-batch fermentation of Rut-C30 and Δ*res2* in glucose (A) and lactose (B). The timepoint of DTT addition is marked with an arrow.

**Additional file 4.** Clusters of differentially expressed genes in RutC30 in four different comparisons.

**Additional file 5.** GO terms of clustered DEGs of RutC30.

**Additional file 6.** Clusters of differentially expressed genes in the delta res2 mutant in four different comparisons.

**Additional file 7.** GO terms of clustered DEGs in the delta res2 mutant.

**Additional file 8.** List of primers used in this study.

## Data Availability

Transcriptomic data can be accessed on the GEO website (https://www.ncbi.nlm.nih.gov/geo/) with the accession number GSE233738.

## Abbreviations

ABC: ATP-binding cassette
DE: Differentially expressed
DEG: Differentially expressed gene
GD/GC: Glucose DTT/glucose control
LC/GC: Lactose control/glucose control
LD/LC: Lactose DTT/lactose control
LD/GD: Lactose DTT/glucose DTT
L2FC: Log2 fold change
GO: Gene ontology
MFS: Major facilitator superfamily
PDA: Potato dextrose agar
RT: Room temperature
TF: Transcription factor
WT: Wild type
