# Supplementary material for: Effect of the res2 transcription factor gene deletion on protein secretion and stress response in the hyperproducer strain Trichoderma reesei Rut-C30
Source: BMC Microbiol. 2023 Nov 30;23:374. doi: 10.1186/s12866-023-03125-z (PMC10687790; doi:10.1186/s12866-023-03125-z)
Supplement: Supplementary file 1 — Additional file 1. Hyphal growth of Rut-C30 and Δres2 grown on different substrates. After 21h, almost all hyphae started branching in glucose and lactose. Germination was slower on lactose and even slower in HEC. [file 12866_2023_3125_MOESM1_ESM.docx]

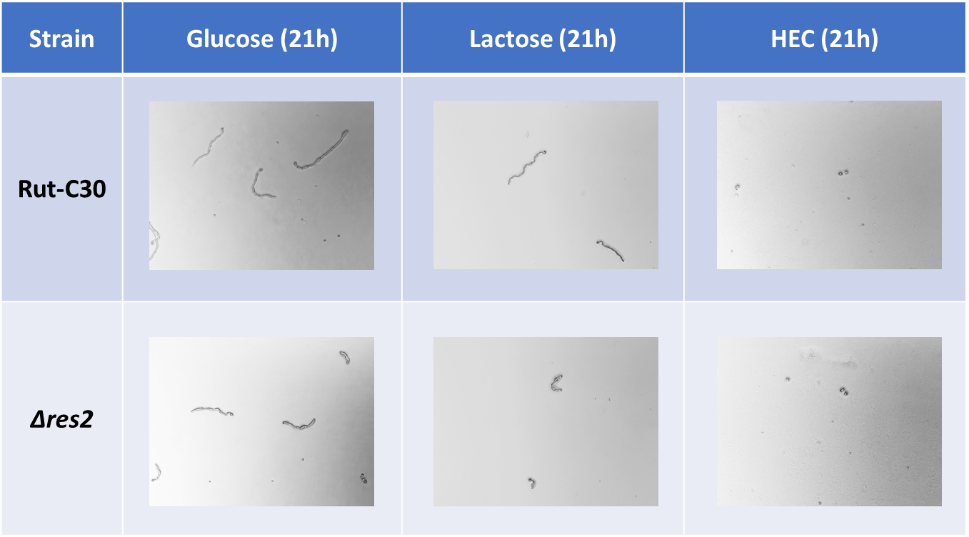


**Additional file 1.** **Hyphal growth of Rut-C30 and Δ*res2* grown on different substrates.** After 21h, almost all hyphae started branching in glucose and lactose. Germination was slower on lactose and even slower in HEC.
