# Supplementary material for: Effect of the res2 transcription factor gene deletion on protein secretion and stress response in the hyperproducer strain Trichoderma reesei Rut-C30
Source: BMC Microbiol. 2023 Nov 30;23:374. doi: 10.1186/s12866-023-03125-z (PMC10687790; doi:10.1186/s12866-023-03125-z)
Supplement: Supplementary file 2 — Additional file 2. Relative gene expression of the secretion stress biomarkers pdi1 and hac1, and the cellobiohydrolase gene cbh1 in Rut-C30 in fed-batch cultures. RNA samples were taken at three timepoints (2h, 4h, 6h) after the addition of DTT. The relative expression is shown with respect to that on glucose at 2h. The error bars indicate standard deviation of two biological replicates. [file 12866_2023_3125_MOESM2_ESM.docx]

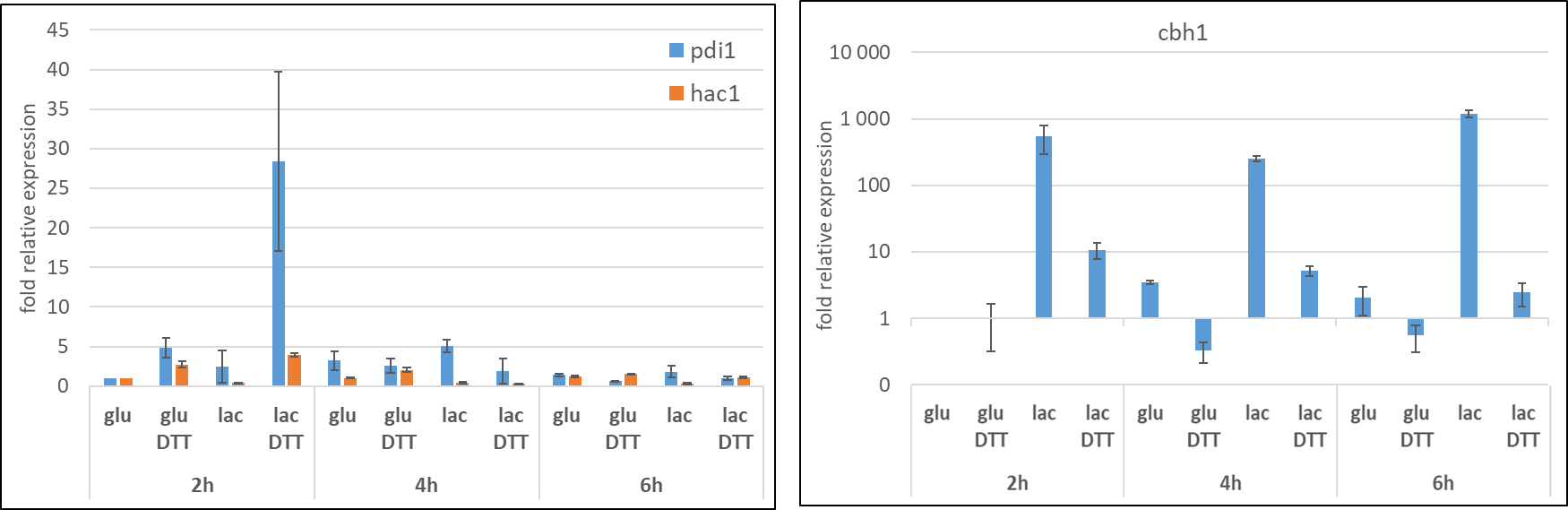


**Additional file 2 : Relative gene expression of the secretion stress biomarkers *pdi1* and *hac1,* and the cellobiohydrolase gene *cbh1* in Rut-C30 in fed-batch cultures.** RNA samples were taken at three timepoints (2h, 4h, 6h) after the addition of DTT. The relative expression is shown with respect to that on glucose at 2h. The error bars indicate standard deviation of two biological replicates.
