# Supplementary material for: Effect of the res2 transcription factor gene deletion on protein secretion and stress response in the hyperproducer strain Trichoderma reesei Rut-C30
Source: BMC Microbiol. 2023 Nov 30;23:374. doi: 10.1186/s12866-023-03125-z (PMC10687790; doi:10.1186/s12866-023-03125-z)
Supplement: Supplementary file 3 — Additional file 3. Protein and biomass profiles of fed-batch fermentation of Rut-C30 and Δres2 in glucose (A) and lactose (B). The timepoint of DTT addition is marked with an arrow. [file 12866_2023_3125_MOESM3_ESM.docx]

| **A**  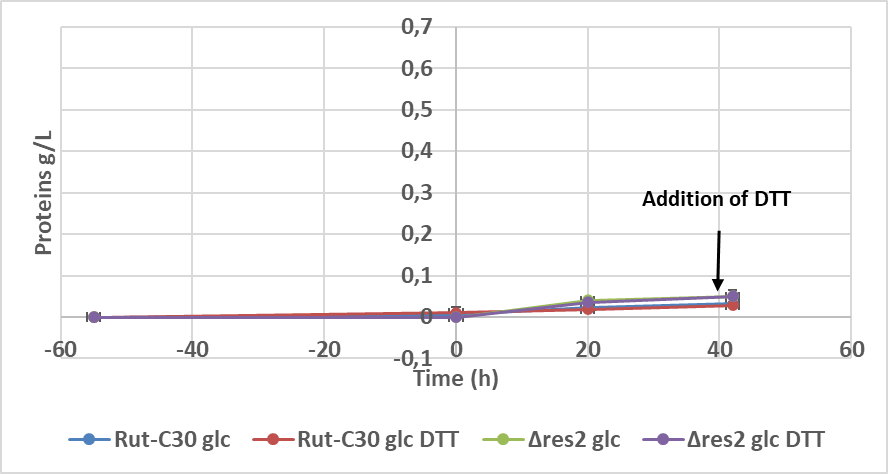 | 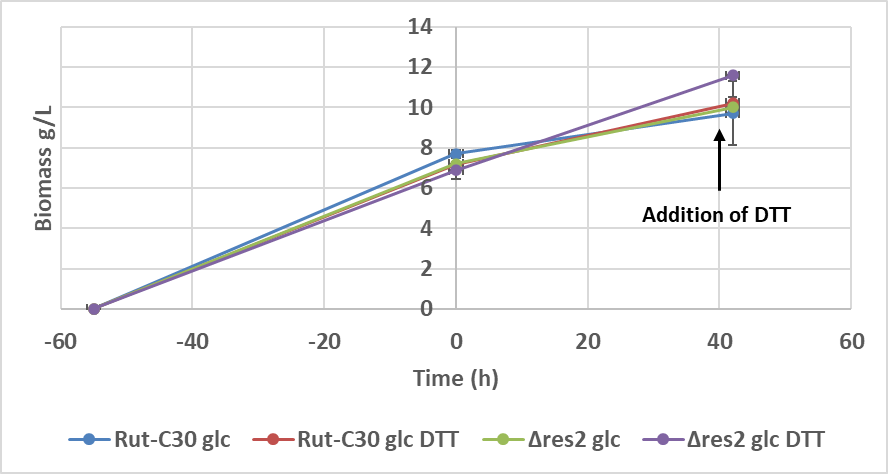 |
| --- | --- |
| **B**  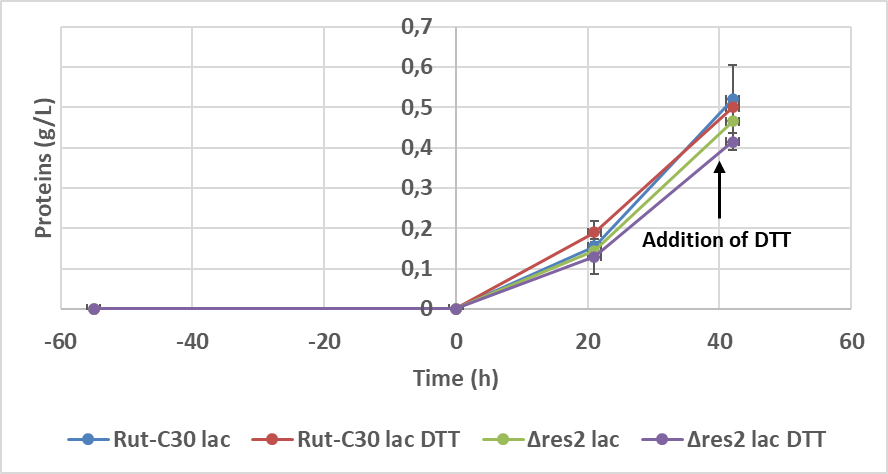 | 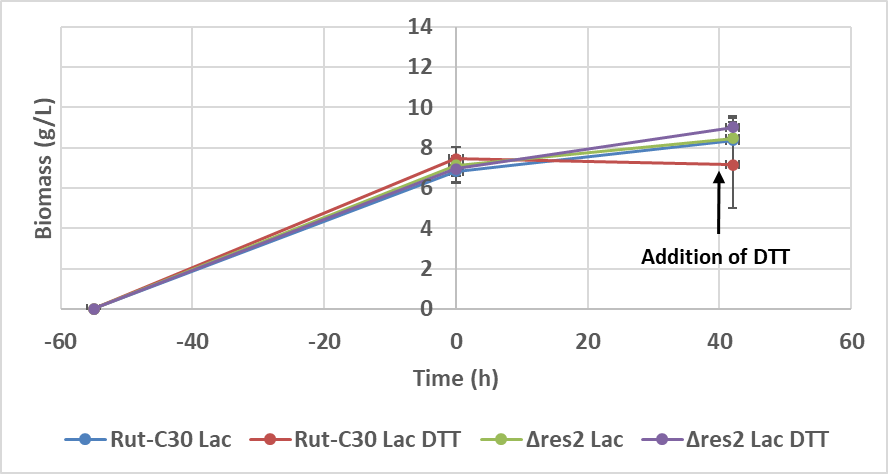 |

**Additional file 3.** Protein and biomass profiles of fed-batch fermentation of Rut-C30 and Δ*res2* in glucose (A) and lactose (B). The timepoint of DTT addition is marked with an arrow.
